# The Collaboration Between Art History and Genetics – An Unlikely Marriage of Disciplines

**DOI:** 10.3389/fpls.2021.757439

**Published:** 2021-11-01

**Authors:** Ive De Smet, David Vergauwen

**Affiliations:** ^1^Department of Plant Biotechnology and Bioinformatics, Ghent University, Ghent, Belgium; ^2^VIB Center for Plant Systems Biology, Ghent, Belgium; ^3^Amarant vzw, Ghent, Belgium; ^4^AP Hogeschool, Antwerp, Belgium

**Keywords:** art, genetics, fruit, vegetable, painting, history

## Abstract

Our fruits, vegetables, and cereal crops stem from a wild ancestor and have undergone major changes through millennia of domestication and selection. There are various ways to reveal plant diversity over time, and one of these is through the combination of art history and genetics (also known as #ArtGenetics). Here, we discuss this approach from the art historian’s point of view and flag the advantages and caveats of such an approach. We also advocate for the development of an integrated, global art database to facilitate such analyses.

Scientists generally receive extra brownie points for interdisciplinary research. Special conferences are organized around it and scholarship applicants are often confronted with an evaluation criterion intended to promote such interdisciplinary research. The reasoning is that creativity is sharpened, and the overall view is broadened when researchers allow insights and results from other disciplines to play a role in their own field of study. In reality, these interdisciplinary studies often turn out to connect fields that were not that far apart to begin with. Nevertheless, a cell biologist who uses insights from biochemistry must be regarded as a fine example of interdisciplinary research. The story becomes more intricate when insights from the alpha sciences are combined with those from the beta sciences. This is the case for research that combines knowledge and methods of art history with those of genetics.

Research based on two disciplines that are so far apart is not self-evident and can only be successful if the research questions cannot be answered successfully in any other way. Authentic interdisciplinary research combines insights and methods from two disciplines –, however, far apart they may be – to tell a single unambiguous story, based on a clear research question. The research question that is central here is: how can we know what our current fruit and vegetables looked like in the past? Interesting additional questions are: where and when were specific crops cultivated? Which (human-driven) migration did they experience? How and why were they incorporated into a culinary culture?

This research question clearly revolves around history. At the root of any complex civilization is the need for food security, which comes in the form of calories. These calories can be obtained through agriculture, animal husbandry, trade, colonization, or conquest. The inability to guarantee food stability inevitably leads to hunger, social and political instability, uprisings, revolution and even the collapse of old civilizations followed by the emergence of new ones. The so-called food historians have been studying these processes since the 1980s and they too employ interdisciplinary research, applying the cultural, economic, environmental, and sociological impact of food and food patterns on human history (Ritchie, 1981; Heiser, 1990; Mintz, 1996; Parasecoli and Scholliers, 2012). Although this discipline will soon be half a century old, we propose a slightly different approach here, in which the history of our food is told through the combination of art history and genetics (also known as #ArtGenetics; Vergauwen and De Smet, 2016, 2017, 2019a,b, 2020).

The question of the exact form and appearance of something in the past, inevitably raises the question of the image. This in turn brings us to art, ranging from (oil) paintings, to mosaic floors, coins, tapestries, murals, pottery, sculpture, architecture, furniture, etc., especially the way in which man shapes his relationship to the world through the image. The uniqueness of the human being itself brings with it a constant occupation with food as expressed in Roman murals with fruit, over Dutch still lifes to Andy Warhol’s soup cans. In all cases, this art is an expression of the relationship between humans and their food. In this way, all art museums in the world can jointly be regarded as the largest database of visual information about fruit and vegetables in a historical perspective.

On the other hand, geneticists have now identified the underlying genes that are responsible for the shape, smell, color and taste of our current crops. As a result, we now know exactly why the tomato is red and the carrot is orange. In the 1930s, Soviet scientist Nikolai Ivanovich Vavilov came up with the idea of looking for wild species that could have served as ancestral variants of our common crops. He followed the logic that a region with a great diversity of wild varieties of the same species could well be the spot where that particular crop originally came from and where it was possibly first bred (Vavilov, 1987).

As a result, it is possible, on an objective scientific basis, to trace the origin of an existing crop and to outline the genetic pathway(s), which have led to the current supermarket varieties through a process of breeding. However, questions that pose quite the challenge are exactly when this happened, by whom, and why. In the absence of additional data sources, geneticists can struggle to infer exactly when successive breeding steps took place and what the consequences were for the appearance of the crop. If we can reconstruct the genetic history of a particular crop – assisted by paleo and archaeogenomics (Kistler et al., 2020) – and we can then plot every appearance of a crop within art through time (chronological) and space (geographic), then all of this can provide us with a very satisfactory picture of its evolution and of the migration of this crop. In doing so, the genotype is linked to the phenotype. These results can then be weighed against the economic, social, political, and ecological aspects of this story that have been studied by the aforementioned food historians for decades. This means that it is possible, at least in principle, to write a complete history of current crops in all its aspects.

In such a case, genetics and art history act as equal partners in a story that is essentially historical in nature. In addition to the ecological, economic, political, and social elements studied by food historians, it is also possible to delve deeper into the artworks themselves and to decipher the symbolic meanings of fruits and vegetables. In her iconography, a painting can prove to us the presence of a pineapple in mid-17th century England (Figure 1A), or the appearance of an orange carrot in Antwerp in the mid-16th century (Figure 1B). However, in some cases the analyses need to go further than iconography, which merely identifies, collects, and describes what is depicted and relates this to textual sources. For example, the piece of vegetable or fruit does not always primarily refer to the actual edible product, but can be a symbolic representation of an abstract concept. This way of thinking was introduced in the first half of the 20th century by Aby Warburg and Erwin Panofsky and is called iconology, which is derived from synthesis rather than scattered analysis and examines symbolic meaning on more than its face value by reconciling it with its historical context (Panofsky, 1939). For an artist or an art historian, this is quite a familiar case to argue, but to a biologist this might sound complicated.

**FIGURE 1:**
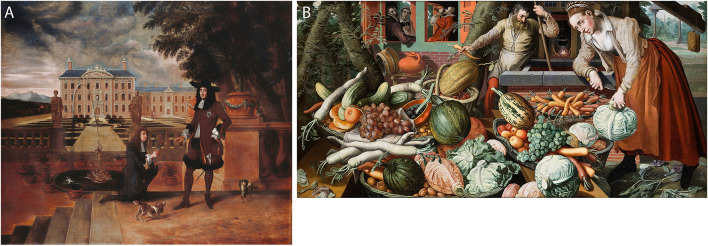
Representative examples illustrating depicted fruits and vegetables. **(A)** Hendrick Danckerts, King Charles II of England, Scotland and Ireland, being given a pineapple by his royal gardener, John Rose, 1675, oil on canvas, Royal Collection. **(B)** Pieter Aertsen, market scene, 1569, oil on canvas, Hallwyl Museum.

This way of looking implies that the presence of a strawberry on a 16th-century painting is not primarily a depiction of a snack that was popular at the time, but rather a reference to the Catholic concept of Mary as a virgin and mother of God. A grape in the hands of Christ in a 17th-century painting may refer to the Savior’s suffering through the blood of the Passion, as explained by the Catholic teaching of transubstantiation where wine (made from grapes) is converted into blood during the Eucharist. Again: this all sounds perfectly reasonable to the art historian, but the biologist will most likely choose not to trust such pictural evidence. The biologist might instead choose to rely exclusively on botanical drawings [nicely illustrated for tomatoes by Andel et al. (2021)], found in scientific treatises on the ground that these were at least produced with the explicit intent to depict the natural world. A database of botanical drawings could be a fascinating tool, but here we are considering an even greater database, which is the whole of art. A biologist might shy away from using artistic images because of their unreliability. That is why collaboration on this point is both necessary and valuable.

It is exactly this tendency toward iconological interpretation, among other things, that has led botanists to distrust the use of art as a valuable source for their own research. They have no way of knowing whether the apple they are looking at on a painting is a true representation of reality or rather refers to a symbolic or metaphysical universe where the apple is a complex symbol that stands for something completely different. Someone who, looking at the apple in Eve’s hand in the garden of Eden, wants to determine in which season the scene exactly takes place, is on very thin ice indeed.

Moreover, art by its very nature has the reputation of being unreliable. After all, we are dealing with paintings and not with photographs. From the biologist’s point of view, there is no way to find out whether the observation of reality was true to nature or not. All of this helps to explain why biologists think twice before including material from art history in their analysis. They don’t trust the available material. At best, an article about a certain crop is accompanied by a work of art, but its use does almost never exceed the level of the illustration. In such a case, the picture is merely decorative, and its inclusion cannot in good faith be regarded as constituting interdisciplinary research. Nevertheless, in an approach that was largely pioneered by Jules Janick from Purdue University, artistic sources have occasionally been incorporated in studies on, for example, (water)melon, cucumber, grapevine, and eggplant (Janick and Paris, 2006; Paris et al., 2006, 2009, 2011, 2012, 2013; Janick et al., 2007; Gago et al., 2009; Renner et al., 2021).

How do we solve this problem? How can we ensure that molecular biologists and botanists understand the value of the iconographic source material and use it in their own analysis? The answer can be found in collaboration. The previously mentioned example of the cell biologist confronted with a problem that is essentially biochemical can only make progress by asking for help from a biochemist. Likewise, we suggest that a geneticist seeks help from an art historian in finding out which art to trust. This cooperation should then take place on a basis of equality, in which both scientists try to transfer the knowledge and methods from their own discipline to the other. Only then can this sort of interdisciplinary cross-pollination be truly fruitful.

To be able to tell this story together with the geneticist, it befalls the art historian to place a filter on the existing source material. The purpose of this critical filter is to separate the usable images from the unusable images by means of historical criticism. To this end, a thorough study of style, materials, preservation, iconography and iconology is necessary and this is beyond the usual competences of the average biologist or geneticist. To give an example, studying a cubist painting provides little or no relevant insights into what a pear might have looked like at the beginning of the 20th century.

Creating the critical filter can be done by way of a few crucial steps. First, what was the ambition and reputation of the artist? This is the easiest place to start. It implies that the art historian is aware of the artist’s ambitions with respect to representing the physical world as he sees it and the extent to which this ambition was considered successful by his contemporaries. In 2020, an exhibition about the work of the Flemish master Jan Van Eyck in the city of Ghent opened under the subtitle “An optical revolution” (Martens et al., 2020). In this way the curators emphasized the artist’s relationship with the observed reality. The presence of glassware and floor tiles from the time of Van Eyck illustrated how faithfully the artist could reproduce what he observed in the physical world around him. Also, in connection with Jan Van Eyck, it can therefore be assumed that an orange from the early 15th century looked exactly like the one Van Eyck depicted on the famous portrait of Giovanni Arnolfini in 1436. In this case, both the ambition and reputation of Van Eyck reveal this master as very reliable when it comes to his depictions of various fruits and vegetables.

Second, are there any other points of comparison? Fruits and vegetables are of course perishable. Obviously, no melon that was grown during the time of the Roman Empire can currently be found on display in a museum. Only Roman art can offer us a glimpse of the external appearance of such melons: *Ars longa, vita brevis*. Nevertheless, the question of reliability can be investigated on the basis of other elements that are present in a painting. They just need to be verifiable today. For example, a 16th-century painting with a melon can also show a musician holding a musical instrument that looks exactly like the ones we keep on display in our museums. A market scene can take place against the background of recognizable buildings, say on the historic marketplace of Amsterdam, with one or two buildings in the background that closely resemble the historical architecture that is still there today. In such cases, there is no reason to believe that perishable products were treated differently by the artist than was the architecture or the musical instruments.

Third, we must point out that there is wisdom in repetition. When a painting shows a fine image of a curious and yet unknown variety, we could regard this as anomalous. Either the artist decided to manipulate the image according to his own imagination for whatever reason he must have had, or he was confronted with a particularly misshapen or somehow defective specimen. However, if we can find additional images with a similar representation of the crop that were created independently of the first, then the issue suddenly merits further study. We might just have come across a variety that has long since disappeared or that can only be found in very specific geographical areas or in botanical gardens.

The more images of fruits and vegetables from all over the world and from all periods in history we can feed into a database, the more accurate the results will be. After all, the collection is only useful if every depicted fruit and vegetable is identified and embedded in a database. Only then can the above-mentioned evolution in time and space be demonstrated. In recent decades, various museums and research institutes have worked hard on digitizing and annotating their own collections. Ideally, one large global database of the art of humanity must be built according to very detailed criteria and with worldwide input and accessibility. The digital collections of various museums, consisting of a wide variety of objects, are already a step in the right direction. There are, however, a lot of obstacles that need to be overcome. First, vast collections remain as yet uncatalogued, let alone digitalized. Second, there is as yet no initiative to unify existing databases or catalogs or to make use of recently developed artificial intelligence-based applications to make a worldwide semantic web of interlocking botanical images in art. Third, many databases or catalogs suffer from mis-identification of depicted species (e.g., Renner et al., 2008; Paris et al., 2012), which in itself can be regarded as a clear argument for collaboration between botanists and art historians. Solving these issues would help science to tell the story of our food and the story of humanity in general.

## Author Contributions

DV and IDS wrote the manuscript. Both authors contributed to the article and approved the submitted version.

## Conflict of Interest

DV was employed by Amarant vzw. The remaining author declares that the research was conducted in the absence of any commercial or financial relationships that could be construed as a potential conflict of interest.

## Publisher’s Note

All claims expressed in this article are solely those of the authors and do not necessarily represent those of their affiliated organizations, or those of the publisher, the editors and the reviewers. Any product that may be evaluated in this article, or claim that may be made by its manufacturer, is not guaranteed or endorsed by the publisher.
